# Ergosterol Peroxide Isolated from Ganoderma lucidum Abolishes MicroRNA miR-378-Mediated Tumor Cells on Chemoresistance

**DOI:** 10.1371/journal.pone.0044579

**Published:** 2012-08-30

**Authors:** Qing-Ping Wu, Yi-Zhen Xie, Zhaoqun Deng, Xiang-Min Li, Weining Yang, Chun-Wei Jiao, Ling Fang, Sen-Zhu Li, Hong-Hui Pan, Albert J. Yee, Daniel Y. Lee, Chong Li, Zhi Zhang, Jun Guo, Burton B. Yang

**Affiliations:** 1 State Key Laboratory of Applied Microbiology in South China (Ministry-Guangdong Province Jointly Breeding Base), and Guangdong Institute of Microbiology, Guangzhou, China; 2 Guangdong Yuewei Edible Fungi Technology Co. Ltd, Guangzhou, China; 3 Sunnybrook Research Institute, Sunnybrook Health Sciences Centre, Toronto, Canada; 4 Department of Laboratory Medicine and Pathobiology, University of Toronto, Toronto, Canada; Albert Einstein College of Medicine, United States of America

## Abstract

Due to an altered expression of oncogenic factors and tumor suppressors, aggressive cancer cells have an intrinsic or acquired resistance to chemotherapeutic agents. This typically contributes to cancer recurrence after chemotherapy. microRNAs are short non-coding RNAs that are involved in both cell self-renewal and cancer development. Here we report that tumor cells transfected with *miR-378* acquired properties of aggressive cancer cells. Overexpression of miR-378 enhanced both cell survival and colony formation, and contributed to multiple drug resistance. Higher concentrations of chemotherapeutic drugs were needed to induce death of miR-378-transfected cells than to induce death of control cells. We found that the biologically active component isolated from *Ganoderma lucidum* could overcome the drug-resistance conferred by miR-378. We purified and identified the biologically active component of *Ganoderma lucidum* as ergosterol peroxide. We demonstrated that ergosterol peroxide produced greater activity in inducing death of miR-378 cells than the GFP cells. Lower concentrations of ergosterol peroxide were needed to induce death of the miR-378-transfected cells than in the control cells. With further clinical development, ergosterol peroxide represents a promising new reagent that can overcome the drug-resistance of tumor cells.

## Introduction

Cancer frequently relapses after chemo-therapy due to the presence of highly proliferative cells as well as tumor stem cells, which are drug resistant in malignant tumors. Some cancer cells can undergo unlimited self-renewal, invade new territory, initiate new tumors, and are resistant to chemotherapy, as a result of deregulated expression of oncogenes and tumor suppressors. Recent studies indicate that the expression of these genes is largely regulated by a subset of RNAs called microRNAs (miRNAs) [1], [2]. Expression of miRNAs is deregulated in cancer and drug-resistant cells.

Over the past few years, microRNAs have emerged as a prominent class of gene regulators [3]. MiRNAs are single-stranded RNAs, 18–24 nucleotides in length and are generated by an RNase III-type enzyme from an endogenous transcript [4], [5]. MicroRNAs function as guide molecules in post-transcriptional gene silencing mainly by partially pairing with the 3′-untranslated region (UTR) of the target mRNAs [6]. By silencing various target mRNAs, miRNAs play key roles in a variety of regulatory pathways, including control of tissue development [7], cell differentiation [8], cell division [9], proliferation [10], migration [11], morphogenesis [12], and apoptosis [13], [14]. Most importantly, miRNAs have been known to play roles in tumor growth [1] and angiogenesis [2], [15].

It has been reported that *miR-378* is expressed in a number of cancer cell lines [16]. Cells transfected with miR-378 express higher levels of vascular endothelial growth factor than the controls [17]. To understand the biological functions of *miR-378*, we generated a *miR-378* expression construct for functional studies and demonstrated that tumor cell line U87 transfected with *miR-378* formed larger tumors and blood vessels [2]. Further studies have indicated that the miR-378 U87 cells acquired aggressive cancer cell properties and became chemo-resistant. In the course of searching for reagents that could overcome this chemo-resistant property, we used the miR-378 expressing U87 cells as a cellular model and screened a large number of potential products from micro-organisms and herbal medicine. We found that the oil-based fraction of *Ganoderma lucidum* could induce the death of miR-378 expressing cells more effectively than in control cells.

**Figure 1:**
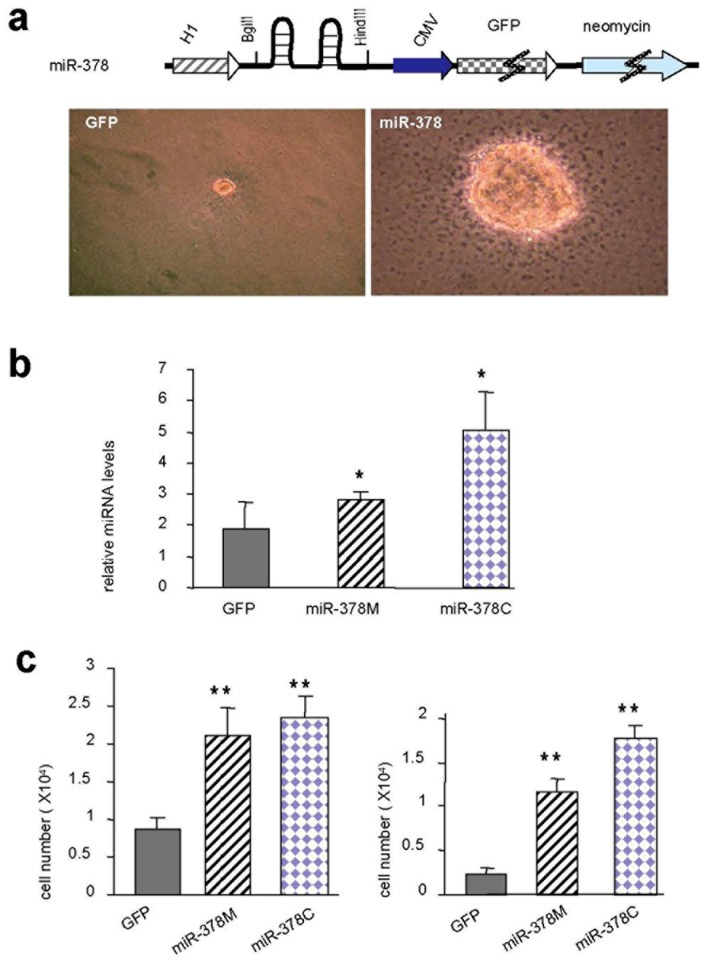
Cancer cells transfected with *miR-378* are C2-ceramide resistant. (a) Upper, diagram of a construct expressing GFP, a neomycin-resistance gene, and two hairpin structures of pre-miR-378. U87 cells transfected with *miR-378* or a control vector were maintained in serum-free conditions in Petri dishes. Cell survival was monitored. Transfection with *miR-378* enhanced cell survival. Lower, in colony formation assays performed in soft agar, miR-378-expressing cells form more colonies with larger sizes. (b) U87 cells transfected with *miR-378* (obtained by flow cytometry thus containing a mix of cell population called **miR-378-mix or miR-378M**) or a control vector (**GFP**) and cells isolated from single colony of miR-378M (**miR-378-col** or **miR-378C**) were cultured in serum-free medium for 1 day. RNAs isolated from the cells were subjected to real-time PCR. miR-378C expressed highest level of miR-378. (c) miR-378M, miR-378C, and GFP cells were cultured in normal medium containing 20 µM (left) and 30 µM (right) C2-ceramide. Cells expressing *miR-378* are resistant to C2-ceramide-induced cell death.

*Ganoderma lucidum* is a traditional Asian medicinal fungus. Its fruit body is called “Lingzhi” in China and “Reishi” in Japan. For hundreds of years, this mushroom has been used as a traditional Chinese medicine. It has been used for the prevention and treatment of many human diseases. In *Ganoderma lucidum*, the major bioactive compounds are polysaccharides, ganoderic acid (triterpene), and adenosine. While the polysaccharides are the major source of its biological activity and the basis of its various therapeutic uses [18]–[21], ganoderic acid also possesses interesting anti-tumour and anti-HIV-1 properties [22], [23]. To date, more than one hundred species of oxygenated triterpenes have been isolated from this fungus, many of which have been identified specifically in this species [24]. There have also been reports that they can inhibit several biological activities including histamine release [25], immunomodulatory activity [26], cytokine production [27], and differentiation-inducing activity [28].

**Figure 2:**
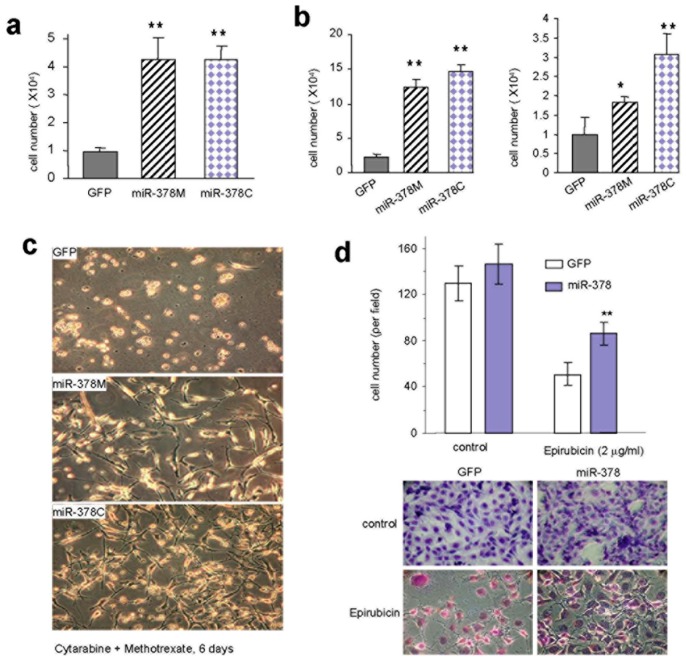
Cancer cells transfected with *miR-378* are resistant to Cytarabine and Methotrexate. (a) miR-378M, miR-378C, and GFP cells were cultured in normal medium containing 56 mM Cytarabine for 3 days. The number of cells was counted and analyzed statistically. The *miR-378* expressing cells exhibited higher rates of survival than the GFP cells. N = 3, ** p<0.01. (b) The cells were treated with Methotrexate at 22 mM for 6 (left) and 8 (right) days. Cells expressing *miR-378* are resistant to Methotrexate-induced cell death. (c) The cells were also treated with combination of Cytarabine and Methotrexate. The combination of both drugs was able to induce cell death at lower concentrations. (d) MDA-MB-231 cells stably transfected with miR-378 or a control vector were treated with Epirubicin (2.0 µg/ml). Cells expressing miR-378 displayed resistance to Epirubicin as compared with the vector control. Typical photos of cell death are shown (lower panel). ** p<0.01.

Traditionally, the fruit body of *Ganoderma lucidum* has been the only part used for medicinal purposes. With improvements in cultivating techniques however, it has been possible to obtain large quantity of spores produced by the fruit body and it has recently been recognized that the spores of *Ganoderma lucidum* possess more potent effect than the fruit body [29]. As a result of their unique components, the spores have been shown to be very effective in disease treatment. We have developed an enzymatic method to digest the sporoderm and obtain large quantities of sporoderm-broken spores to isolate the oil-based fraction. We found that the oil-based fraction can induce cancer cell death [30]. In this study, we investigated the role of the oil-based fraction and the biologically active components in inducing death of the aggressive cancer cells. We also purified the biologically active components and found that the molecule, ergosterol peroxide could effectively induce death of aggressive cancer cells, overcoming the resistance to multiple drugs.

**Figure 3:**
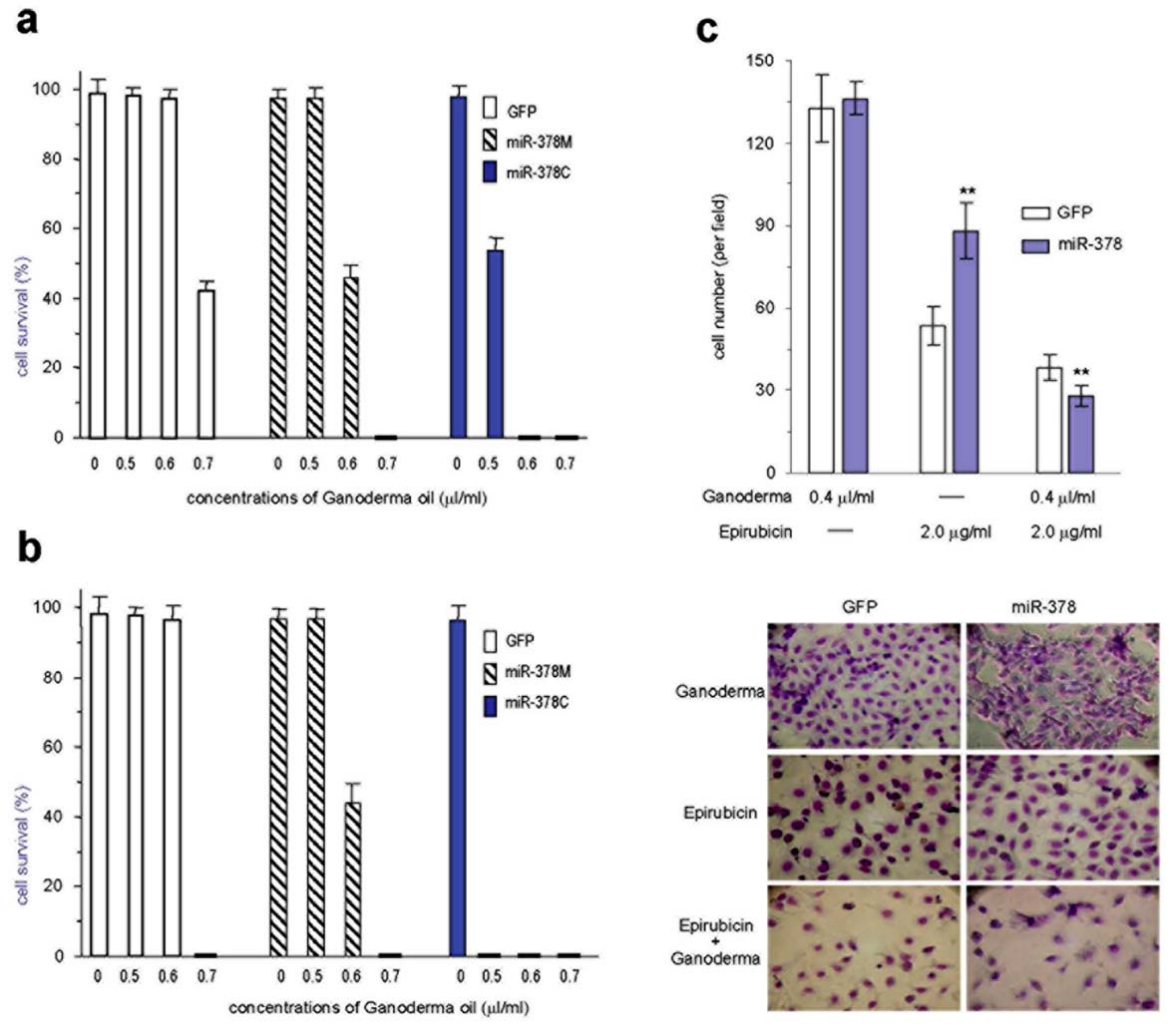
Ganoderma oil exerts stronger effect on inducing death of the cencer cells. (a-b) The GFP, miR-378M, and miR-378C cells were cultured in normal medium treated with different concentrations of Ganoderma oil (prepared form the standard protocol) as indicated for 24 hours (a) or 48 hours (b). The effects of Ganoderma oil on cell death were miR-378C > miR-378M > GFP. (c) MDA-MB-231 cells stably transfected with miR-378 or a control vector were treated with Epirubicin (2.0 µg/ml) and combined with or without Ganoderma oil 0.4 µl/ml. The miR-378-transfected cells were less sensitive to Epirubicin than the control cells. Combination with Ganoderma oil significantly increased the sensitivity of the miR-378 cells to Epirubicin. Typical photos of cell death are shown (lower panel). ** p<0.01.

## Methods

### Construct Generation

A miRNA construct expressing *miR-378* was designed by our lab and generated as previously described [2], [31]. This plasmid contains a Bluescript backbone for duplication of the plasmid, a human H1 promoter driving two pre-miR-378 units, and a CMV promoter driving the expression of a green fluorescent protein (GFP) for monitoring the expression of the plasmid. This plasmid has been used successfully in many reports from our lab. The control plasmid was the same except the pre-miR-378 sequence was replaced with a non-related sequence (atacagtactgtgataactgaagtttttggaa**aagctt**tagttattaa), serving as a mock control, which also contained a GFP unit for monitoring transfection efficiency.

### RT-PCR

The levels of miRNA were assessed by real-time PCR using methods previously described [32], [33]. In brief, total RNA was extracted from ∼1×10^6^ cells using mirVana™ miRNA Isolation Kit (Ambion). The isolated RNA was subjected to reverse transcription. The synthesized cDNA was used as a template for successive PCR with the QuantiMir RT Kit (Qiagen). The primers miR-378N (5′agatctagggctcctgactcc) and miR-378C (5′aggccttctgactccaagtcc) were used for detection of miR-378. The primers used as real time PCR controls were human-U6RNAf (5′ gtgctcgcttcggcagcacatatac) and human-U6RNAr (5′ aaaaatatggaacgcttcacgaatttg).

**Figure 4:**
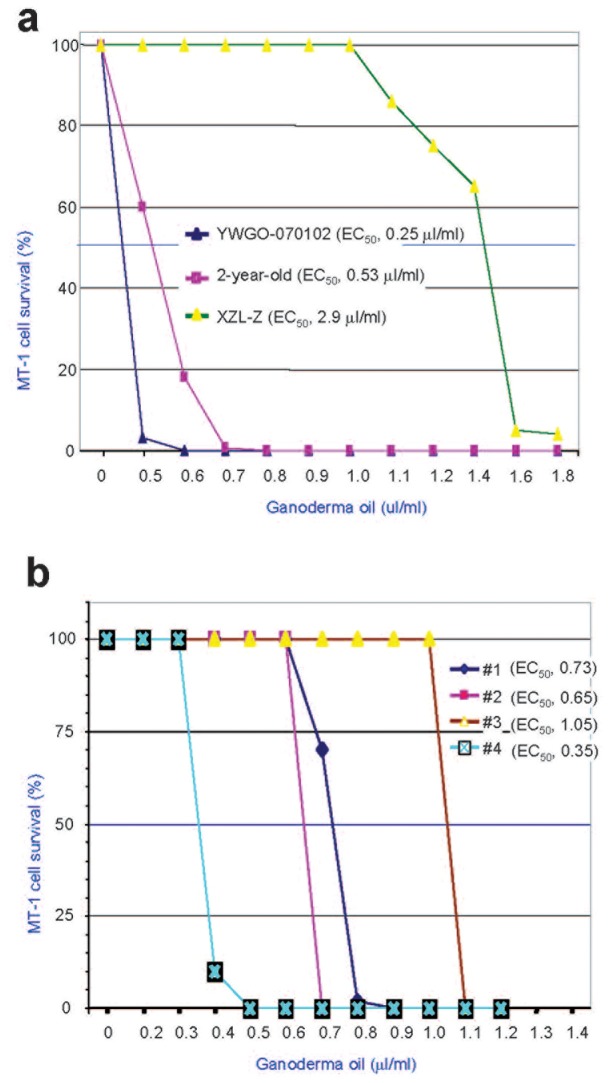
Effects of Ganoderma oil from different sources on tumor cell death. (a) Breast carcinoma cells (MT-1) were treated with Ganoderma oil prepared from the standard protocol (WYGO-070102), 2-year-old (maintained at room temperature) Ganoderma oil prepared from the standard protocol, and a sample of Ganoderma oil obtained from a different source (XZL-Z). Analysis of the half maximal effective concentration (EC_50_) indicated that the Ganoderma oil prepared from the standard protocol produced the best result in inducing tumor cell death, followed by the 2-year-old product and the sample from a different source. (b) MT-1 cells were treated double-blindly with Ganoderma oil obtained from different sources. EC50 was obtained as indicated.

### Isolation of Ganoderma Oil

Spores of *Ganoderma lucidum* were obtained from the fruit body cultivated on logs of wood of a *Ganoderma lucidum* farm in the Dabie Mountain in An-Hui Province. The spores were treated with enzymes released from the spores by germination for different periods of time using the GMP manufacturing facilities of Guangdong Yuewei Edible Fungi Technology Co. Ltd. The oil-based fraction in the treated spores was isolated by Supercritical Fluid Extraction (SFE). Carbon dioxide (CO_2_) was used as the supercritical fluid (SCF) due to its non-toxicity, low critical parameters (31.1°C, 73.8 bar), and low cost. At this critical point, the carbon dioxide is neither a gas nor a liquid and is described as intermediate to the two extremes. This phase of carbon dioxide retains solvent power similar to liquids and transport properties similar to gases. When the supercritical fluid of carbon dioxide is mixed with Ganoderma spores, the soluble oil- based fraction was transferred to the carbon dioxide fluid. By changing the temperature and pressure, we were able to isolate different fractions of the oil-based substance. As part of the standard protocol, we conducted the extraction at a temperature of 40 °C±5°C, and a pressure of 25 MPa ±1 MPa. The carbon dioxide fluid moved at a velocity was 175 L/h±15L/h. The Separating Pressure was 6 Mpa ±1 Mpa, while the Separating Temperature was 45 °C±2 °C.

### Purification of Biologically Active Fractions

Column fractionation was the process used to purify the biologically active ingredient of Ganoderma. Ganoderma oil (210 g) was mixed with petroleum ester (250 ml), also known as benzine, and silica gel (420 g, 160–200 mesh). The mixture was maintained at 60°C to evaporate the solvent petroleum ester. In the meantime, 6.3 kg silica gel was packed into a column of 15×70 cm (d×h). The dried sample mixture of Ganoderma oil and silica gel was applied as a top layer on the surface of the silica gel bed. After careful application of the sample, the column was eluted with four bed volumes of chloroform at an elution speed of 25 ml/min. Each collection contained 500 ml. The column was then eluted with three bed volumes of ethyl ethanoate (25 ml/min, 500 ml for each collection).

After evaporation of the solutions, both elutes from chloroform and ethyl ethanoate were combined, getting 132 g of the chloroform elution and 78 g of the ethyl ethanoate elution. We assessed the activities of both collections in inducing cancer cell death and found that the ethyl ethanoate elute had high capacity in inducing cancer cell death.

The 78 g elute was diluted with petroleum ester and mixed with 156 g silica gel. The mixture was subjected to evaporate at 60°C till dried. It was then applied to a silica gel column (6×100 cm) packed with 1.5 kg silica gel. The column was eluted with different mixture of eluent as follows: 10 liters of the mix of petroleum ester and ethyl ethanoate (at the ratio of 20∶1), 10 liters of petroleum ester and ethyl ethanoate (9∶1), 10.5 liters of petroleum ester and ethyl ethanoate (8∶2), 10 liters of petroleum ester and ethyl ethanoate (7∶3), 9.5 liters of petroleum ester and ethyl ethanoate (5∶5), 7.5 liters of ethyl ethanoate, at the elution speed of 15 ml/min, 500 ml for each collection.

Each collection was analyzed on Thin Layer Chromatography. Collections showing similar pattern of movements were combined. A total of 13 different samples were obtained labeled L1, L2-1, L2-2, L3-1, L3-2, L4-1, L4-2, L5-1, L5-2, L5-3, L5-4, L6-1, and L6-2.

Based on the anti-cancer cell activities of each fraction, 1 g of L4-2 fraction was diluted with acetone and mixed with 1.5 g silica gel. The mixture was subjected to evaporation at 60°C to remove acetone. The dried sample was applied to a silica gel column (2.5×50 cm containing 50 g silica gel). The column was eluted with 680 ml of eluent containing petroleum ester and acetone (at the ratio of 8∶2) at the elution speed of 20 ml/15 min. Each collection contained 20 ml. The 34 collections were subjected to evaporation in a rotary evaporator (Type RE3000A, Shanghai Yarong Biochemistry Instrument Factory).

Based on the anti-cancer cell activities of each sample, the collection No 21 (300 mg) was subjected to further purification by Reversed-Phase HPLC using Agilent HPLC1200 (product of Agilent Technologies Inc). This was conducted using the column YMC-C18 (10×250 mm, 10 µm), and eluted with a mixture of methanol and water (at the ration of 96∶4 or 96% methanol) at the elution speed of 4 ml/min. After 60 min, a fraction GL421 was obtained, which contained 32 mg elute. NMR was used to identify the substance’s molecular structure and molecular weight.

**Figure 5:**
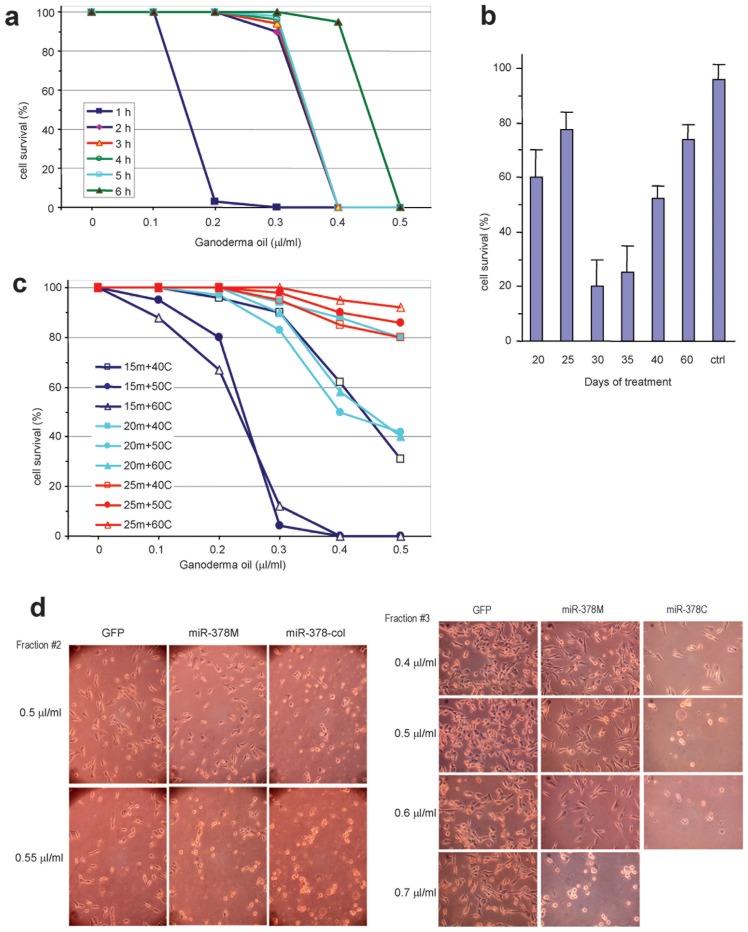
Effects of Ganoderma oil prepared using different protocols on tumor cell death. (a) Ganoderma spores were subjected to oil extraction using the standard protocol, in which the spores were maintained under the standard pressure for 1–6 hours. The effects of the oil on tumor cell death were tested. (b) Ganoderma spores were digested with spore-released enzymes for different periods of time as indicated, followed by oil extraction with the standard protocol. The effects of the oil on tumor cell death were tested. (c) Ganoderma spores were subjected to oil extraction under different temperature and pressure. The effects of the oil on tumor cell death were tested. “m” means the pressure Mpa while “C” means degree of the temperature. (d) Typical results of the effects of “15m+50C” (Fraction #2) and “15m+60C” (Fraction #3) on tumor cell death are shown.

### EM Picture

Cell cultures treated with or without Ganoderma oil were harvested as pellets. The pellets were immersed in 2.5% glutaraldehyde in 0.1 M cacodylate buffer, pH 7.4 for 2 hours to fix the cells. The pellets were washed in 0.1 M cacodylate buffer with 4% sucrose, pH 7.4, for three times, 5 minutes each. The samples were subjected to a second round of fixation in 1% osmium tetroxide in 0.1 M cacodylate buffer, pH 7.4, for 1 hour, followed by three washes, 5 minutes each, in 0.1 M cacodylate buffer, pH 7.4. The samples were stained with 1% uranyl acetate dissolved in 25% ethanol (or water) for 1 hour, followed by a process of dehydration in the sequence as follows: 50% ethanol, 70% ethanol, 90% ethanol, and 95% ethanol (10 minutes each), three times in 100% ethanol (EM grade) (10 minutes each), and three times in acetonitrile (10 minutes each). After dehydration, the samples were infiltrated and embedded in 50/50 acetonitrile/spurr’s resin for 1 hour, followed by incubation in 100% spurr’s resin for 1.5 hours. This step was repeated once. The samples were then oriented into mould and additional resin was added. The sample-containing moulds were placed in at oven at 60°C for 24 hours. They were then sectioned for electron microscopic examination and photographed.

**Figure 6:**
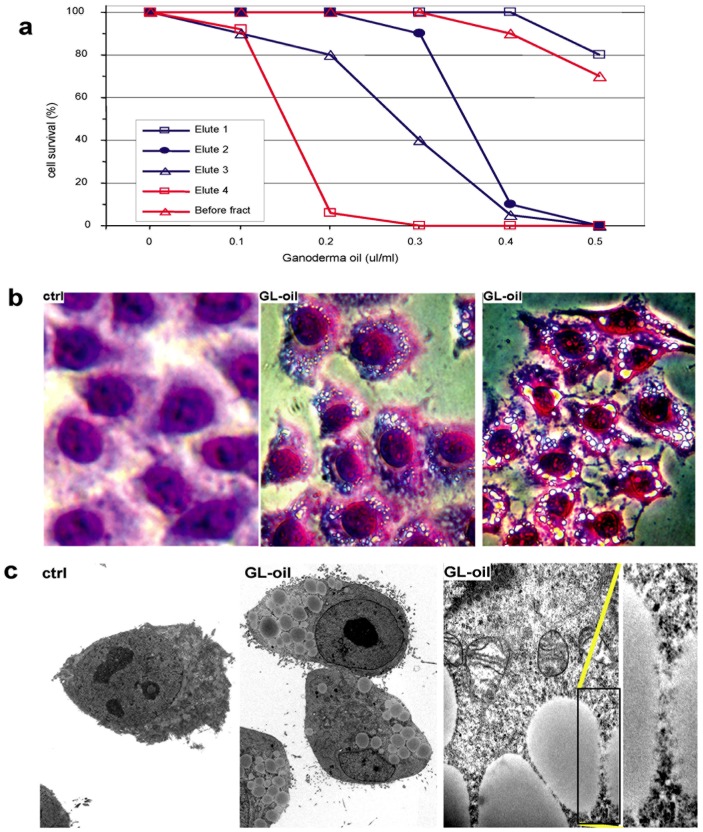
The effects of biologically active components on tumor cell death. (a) Biologically active fractions were eluted with different chemicals, followed by analysis of their effects on tumor cell death. The fraction eluted by methanol (Elute 4) produced the greatest activity, while Elute 1 and the sample before fractionation (Before fract) displayed the lowest activity on inducing tumor cell death. (b) The MT-1 cells treated with or without Ganoderma oil were fixed, and stained with the Diff-Quik-Stain kit, followed by microscopic examination. A great number of vacuoles were detected in the cells treated with Ganoderma oil. (c) The Ganoderma oil treated cells were subjected to electronic microscopic examination. Large vacuoles could be seen clearly.

### Cell Survival Assay

To test drug resistance of the miR-378-transfected cells and effects on Ganoderma oil in inducing cancer cell death, we performed cell survival assays. Cells (1×10^4^ cells/well) were seeded on 12-well tissue culture plates in DMEM containing 10% FBS. The cells were allowed to adhere and spread on the plates for a period of two hours. Chemotherapeutic drugs were added to the culture at the concentrations indicated in each figure. The cultures were treated with the drugs for different periods of times. The cultures were harvested and cell number was counted using trypan blue staining as previously described [34]. For the treatment with Ganoderma oil, the oil was pre-dissolved in DMSO at a concentration of 2% as a stock solution. The stock solution was added to the cultures two hours after cell inoculation at the concentrations indicated in each figure. Cell viabilities were measured 24 or 48 hours after the treatment.

**Figure 7:**
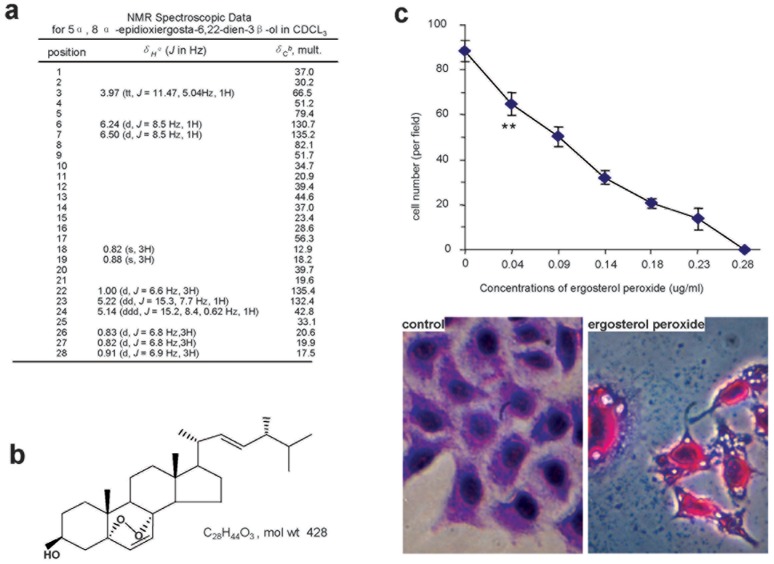
The effects of ergosterol peroxide on tumor cell death. (a) NMR analysis of the structure of the candidate. (b) Molecular structure, formula and molecular weight of ergosterol peroxide. (c) The MT-1 cells treated with or without ergosterol peroxide were fixed, and stained with the Diff-Quik-Stain kit, followed by microscopic examination and counting of cells survived. Induction of breast cancer cell death by ergosterol peroxide was concentration dependent (Upper). ** 0<0.01, n = 3. Typical vacuoles were detected in the cells treated with ergosterol peroxide (Lower).

### Colony Formation in Soft Agarose Gel

To analyze aggressive activity of the miR-378-transfected cells, colony formation assays were performed. It was assessed using a method described previously [35]. In brief, 10^3^ cells were mixed in 0.3% low-melting agarose in DMEM supplemented with 10% FBS and plated on 0.66% agarose-coated 6-well tissue culture plates. Colony formation and growth were monitored every other day. Four weeks after cell inoculation, colonies were examined under a light microscope and photographed.

### Statistical Analysis

The results (mean values ± SD) of all the experiments were subjected to statistical analysis by *t*-test. The level of significance was set at p<0.05 and p<0.01.

**Figure 8:**
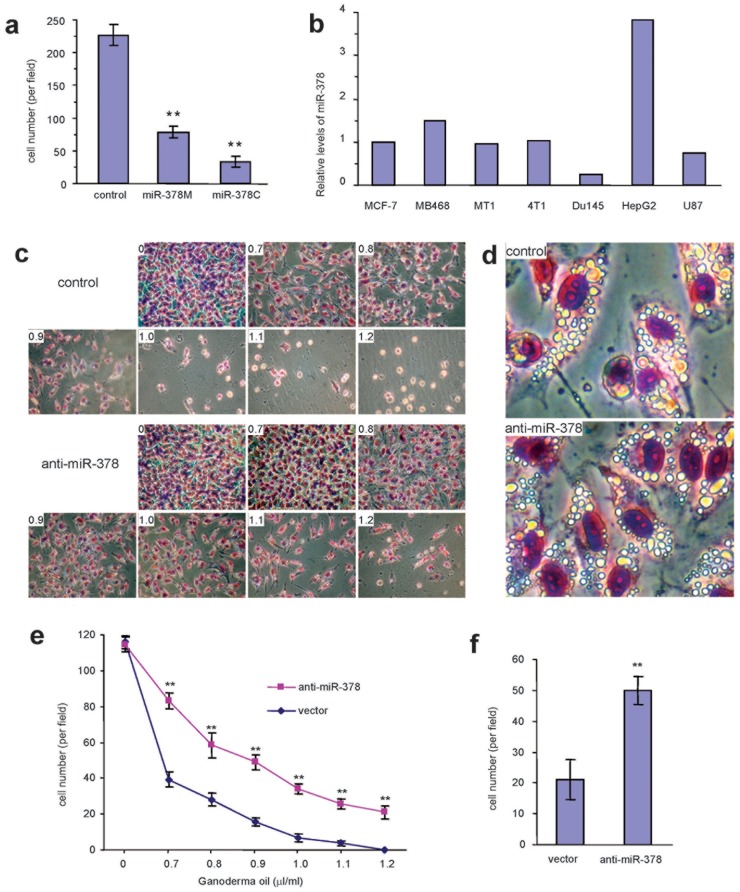
Effect of miR-378 on Ganoderma oil and ergosterol peroxide induced cell death. (a) GFP, miR-378M, and miR-378C cells were treated with ergosterol peroxide at the concentration of 0.23 mM for 48 hours. The chemical exhibited strongest activity on inducing death of miR-378C cells, followed by miR-378M and GFP cells. ** 0<0.01, n = 3. (b) RNAs were isolated from a number of cancer cell lines and subjected to real-time PCR analysis for miR-378 expression. The human hepatocellular carcinoma cells HepG2 express the highest levels of miR-378 among the cell lines tested. (c) Human hepatocellular carcinoma cell line HepG2 was transfected with anti-miR-378 or a control vector, followed by treatment with Ganoderma oil. Transfection with anti-miR-378 construct decreased the sensitivity of the cells to Ganoderma oil induced cell death. (d) Typical morphology of the cells treated Ganoderma oil is shown. (e) After the treatment with Ganoderma oil, the number of the cells was counted. Significant differences were seen in both groups of cells treated with different concentrations of Ganoderma oil. ** p<0.01. (f) The cells were also treated with ergosterol peroxide. Antagonizing endogenous miR-378 decreased the sensitivity of the cells to the induced cell death by Ganoderma oil. ** p<0.01.

## Results and Discussion

### Cell Survival Affected by *miR-378* Expression

We have previously demonstrated that U87 cells expressing miR-378 form significantly larger tumors and tumor blood vessels than cells transfected with a control vector [2]. In this study, we used the U87 cell model to study the effect of miR-378 on drug-resistance. We tested whether the miR-378-transfected cells were more aggressive using a colony formation assay performed on soft agar. We found that more colonies with larger sizes were formed by the miR-378-expressing cells compared with the control (Fig. 1a).

To examine whether or not the cells that formed colonies were those that expressed high levels of miR-378, we isolated cells from the single colonies (referred to as miR-378col or miR-378C). Analysis of miRNA levels indicated that the cells isolated from the single colony expressed significantly higher levels of *miR-378* than cells transfected with *miR-378*, which were selected with flow cytometry thus containing a mix of cell population (referred to as miR-378-mix or miR-378M), or with a control vector (Fig. 1b).

We tested whether or not miR-378 could block the effect of the apoptosis-inducing agent C2-ceramide and observed that U87 cells transfected with *miR-378* exhibited reduced cell death compared with control cells after treatment with C2-ceramide (Fig. 1c, Fig. S1). Our results indicated that cells expressing miR-378 could not only grow faster, but also survive better than the control cells under the treatment of the cell-death-inducing chemical C2-ceramide.

### Cells Expressing *miR-378* are Resistant to Drug-induced Death

It has been reported that cancer cells are drug-resistant [36]. We tested the effects of a number of chemicals and chemotherapeutic drugs on cells expressing miR-378. MiR-378M, miR-378C, and GFP cells were cultured in normal medium treated with Cytarabine at 56 mM for 1 or 3 days. After one day treatment, the miR-378-transfected cells displayed decreased spreading, compared to the GFP-transfected cells (Fig. S2a). Three days after drug addition, the miR-378 cells retained a similar phenotype to the one day treatment. However, the GFP cells showed extensive cell death. A total count of the live cells indicated that miR-378 expression decreased cell death significantly when compared with cells transfected with GFP (Fig. 2a). The cells were also incubated with the drug at a concentration of 40 mM for 5 days. Similarly, miR-378 expression decreased cell death significantly compared with the control (Fig. S2b).

We also treated the miR-378 and GFP cells with Methotrexate at 22 mM for 6 and 8 days. Cells expressing miR-378 were resistant to Methotrexate-induced cell death (Fig. 2b). As well, the miR-378 cells showed less adhesion and enhanced elongation compared with the GFP cells (Fig. S3). The miR-378 and GFP cells were also treated with a combination of Cytarabine and Methotrexate, which resulted in enhanced cell death (Fig. 2c). Thus, our results are consistent with previous reports that cancer cells are drug-resistant. Our results also suggest that the miR-378-transfected cells possessed a drug-resistant property.

The hallmarks of malignant cancer include increased self-renewal, survival ability, and drug resistance. When miR-378 was transfected into the human brain tumor cell line U87, all of these properties were enhanced. In soft agarose gel, the miR-378-transfected cells formed larger colonies than the vector-transfected cells. In serum-free conditions, the miR-378 cells survived better than control cells. In addition, the miR-378 cells demonstrated resistance to cell death induced by different drugs. With these properties, the miR-378 cells can serve as an ideal model for characterizing malignant cancer cells.

We also tested the effects of miR-378 on drug resistance using a different cancer cell line. Human breast carcinoma cell line MDA-MB-231 was stably transfected with miR-378 or a control vector. The cells were treated with Epirubicin (2.0 µg/ml), a chemotherapeutic drug routinely used for treating breast cancer. Cells expressing miR-378 displayed significant resistance to Epirubicin compared with the control cells (Fig. 2d). These results confirmed that miR-378 plays an important role in resistance to drug induced cell death. The effects of this miRNA on other types of cancer cells await further investigation.

### Effect of *Ganoderma lucidum* on Tumor Cell Survival

Due to the toxicity and side effects of chemotherapeutic drugs, more and more cancer patients have turned to herbal medicine for complementary treatment. *Ganoderma lucidum* is a medicinal mushroom that is widely used for this purpose. It has been reported that the spores of *Ganoderma lucidum* are more potent than its fruit body [29]. Indeed, we have previously reported that extracts from the fruit body of *Ganoderma lucidum* can induce tumor cell death [37]. It has been reported that the spores of *Ganoderma lucidum* are more potent than its fruit body [29], since the spores contain unique components in their lipid fraction, which can induce tumor cell death powerfully [30]. We have extracted this lipid fraction, also known as Ganoderma oil, using high pressure carbon dioxide. The GFP, miR-378M, and miR-378C cells were cultured in normal medium treated with different concentrations of Ganoderma oil. We found that treatment with Ganoderma oil induced cancer cell death. Interestingly, the GFP-transfected cells showed only partial cell death at a concentration of 0.7 µl oil per ml per well, while the miR-378M cells showed complete cell death at this concentration (Fig. 3a, Fig. S4). At a lower concentration (0.6 µl/ml), little cell death was detected in the GFP cells. However, the miR-378M cells showed partial cell death, while the miR-378C cells showed complete cell death. These results suggested that the cells most resistant to chemotherapeutic drugs (resistance: miR-378C > miR-378M > GFP) were also the most sensitive to cell death induced by Ganoderma oil (sensitivity: miR-378C > miR-378M > GFP). With longer incubation periods, Ganoderma oil induced progressively greater cell death and sensitivity to cell death (Fig. 3b, Fig. S5).

We then tested the effects of Ganoderma oil combined with chemotherapeutic drug treatment in the human breast carcinoma cell line MDA-MB-231. The cells were stably transfected with miR-378 or a control vector followed by treatments with Epirubicin (2.0 µg/ml), with or without Ganoderma oil. While the miR-378-transfected cells were less sensitive to Epirubicin than the control cells, combination with Ganoderma oil (0.4 µl/ml) significantly increased the sensitivity of the miR-378 cells to Epirubicin (Fig. 3c). It was noted that at 0.4 µl/ml, Ganoderma oil displayed a minimal effect on MDA-MB-231 cells. When combined with Epirubicin, Ganoderma oil increased Epirubicin-induced cell death of the miR-378-transfected cells (Fig. 3c).

Currently, there is no drug that can effectively induce death in tumor stem cells, nor one that can overcome the drug-resistant property of malignant cancer cells. The oil-based fraction of *Ganoderma lucidum* can not only induce cell death of cancer cells (miR-378M and miR-378C), but can also do so more effectively than mock cells. This is opposite to the typical effect of chemotherapeutic treatments. In order to kill the more aggressive miR-378-transfected cells, higher drug concentrations were needed than that to kill the control cells. This is not surprising as aggressive cancer cells develop drug-resistance very rapidly. However, significantly lower concentrations of Ganoderma oil were needed to kill the aggressive cancer cells than to kill the mock tumor cells. In other words, the more aggressive cancer cells were more sensitive to Ganoderma oil than the mock-transfected cells. Patients who incorporate Ganoderma oil as a supplementary treatment frequently reported favorable outcomes. Some patients have a delay in cancer recurrence, have improved quality of life, or even complete recovery from cancer. Nevertheless, the sensitivity to Ganoderma oil treatment appears to vary across different cancer cells. For instance, Jurkat leukemia cells seemed to be more sensitive to Ganoderma induced cell death than MT-1 breast carcinoma cells, a very metastatic cell line in nude mice [38]. Brain tumor cells U87 (obtained from ATCC, Rockville, MD), appeared to be less sensitive than MT-1 cells. Further studies are required to investigate these differences in sensitivity to Ganoderma oil. It is necessary to test the effects of Ganoderma oil in different cancer cell lines, since different types of cancer cells may respond differently to Ganoderma oil.

### Effects of Ganoderma Oil from Different Preparation on Tumor Cell Death

Ganoderma oil obtained from different sources was used in our studies. In addition, the effect of Ganoderma oil was also tested on different tumor cell lines. As above, MT-1 cells were treated with Ganoderma oil prepared from the standard protocol (WYGO-070102) and a sample of Ganoderma oil obtained from a different source (XZL-Z). We found that WYGO-070102 was significantly more effective in inducing tumor cell death than XZL-Z (Fig. 4a, Fig. S6a). The effects of WYGO-070102 and XZL-Z were also tested on lymphoma cells (Jurkat cells obtained from ATCC). Similarly, WYGO-070102 induced cell death at much lower concentrations than XZL-Z (Fig. S6b), although higher concentrations were required to induce MT-1 cell death (Fig. S6a).

We then analyzed the stability of Ganoderma oil in inducing tumor cell death. MT-1 breast carcinoma cells were treated with WYGO-070102, ZXL-Z, and WYGO-050202 that had been stored at room temperature for two years. Previous experimentation had indicated that Ganoderma oil was relatively stable. After being stored for two years, WYGO-050202 was still able to induce tumor cell death more effectively than ZXL-Z (Fig. 4a). These results suggested that the oil-based fraction was more stable than the polysaccharide fraction (data not shown). The effects of Ganoderma oil on tumor cell death were further analyzed with Ganoderma oil from different sources. In a double-blind study, MT-1 cells were treated with Ganoderma oil obtained from different sources. We found that Ganoderma oil obtained from different sources were significantly different in inducing breast cancer cell death (Fig. 4b).

### Isolation of Highly Active Ganoderma Oil

We designed different methods to isolate Ganoderma oil. The Ganoderma spores were treated with enzymes released by the germinating spores for 30 days, and the Ganoderma oil was extracted with the standard protocol for different periods of time. We found that the oil extracted in the shortest period of time (1 hour) exhibited the strongest effect on inducing cancer cell death (Fig. 5a). On the other hand, the oil extracted in the longest period of time (6 hours) exhibited the weakest activity in inducing cancer cell death. Oil extracted for 2, 3, 4, and 5 hours showed a similar effect in inducing cancer cell death (Fig. 5a). The oil extracted in the shortest period of time was expected to have a relatively low molecular mass, suggesting that the smaller molecules in the oil may have played a key role in inducing cancer cell death.

Ganoderma spores were digested with enzymes released from the spores for different periods of time, followed by oil extraction as described above. The effects of the oil on tumor cell death were tested. We found that spores treated with the spore-released enzymes for 30 days were the most effective in inducing cancer cell death (Fig. 5b). Since treatment for 30 days produced better results than treatment for 20–25 days, treatment periods may have led to the synthesis of new molecules, possibly playing an important role in inducing cancer cell death. However, after extended treatment periods, some biologically active molecules may have decomposed and decreased the effectiveness of cancer cell death induction. Thus, controlling treatment times will be critical in producing the best agents for cancer treatment.

Ganoderma spores treated with the spore-released enzymes for 30 days were subjected to oil extraction under a combination of different temperatures and pressures. It was expected that smaller molecules would be extracted under a lower temperature and pressure, while larger molecules could be extracted with a higher temperature and pressure. The experiments showed that Ganoderma oil extracted at a pressure of 15 Mpa and 50–60 degrees Celsius displayed the greatest ability in inducing cancer cell death (Fig. 5c). Typical results of “15m+50C” and “15m+60C” treatments on tumor cell death are shown (Fig. 5d). Oil extracted at 15 Mpa with 40 degrees, 20 Mpa with 50 degrees, or 20 Mpa with 60 degrees had higher activities in inducing cancer cell death than the other treatments. Oil extracted in the following conditions: 20 Mpa with 40 degrees, 25 Mpa with 40 degrees, 50 degrees, and 60 degrees had the lowest ability in inducing cancer cell death (Fig. 5c).

### Purification of Anti-cancer Molecule

Our results above indicated that there were some components in Ganoderma oil that were more powerful in inducing cancer cell death than others. We designed a protocol to isolate the biologically active components from the Ganoderma oil using silica gel columns. After application of the oil to the column, the column was eluted with four different eluents: (1) petroleum ether, (2) 95% petroleum ether mixed with 5% acetone, (3) 90% petroleum ether mixed with 10% acetone, and (4) methanol. The resulting eluate was used to treat the cancer cell cultures. The experiments indicated that the Methanol fraction produced the best outcome in inducing cancer cell death, followed by the others as follows: (4)>(3)>(2)>(1) = control (Fig. 6a).

Careful examination of the cells treated with Ganoderma oil showed that a large number of vacuoles could be seen in the cytoplasm of the cells, which were not seen in the cells treated with DMSO. The cells treated with or without Ganoderma oil were then fixed, and stained with a cell staining kit (Diff-Quik Stain Kit, Dade Behring Inc, Newark, USA), followed by microscopic examination. A great number of vacuoles were detected in the cells treated with Ganoderma oil (Fig. 6b). To examine the vacuoles in greater detail, we examined the cells treated with or without Ganoderma oil by electron microscopy. We detected large vacuoles in the cells treated with Ganoderma oil (Fig. 6c).

After a series of column fractionations and Reversed-Phase HPLC purifications, we obtained a reagent of more than 95% purity. Identification of the sample with NMR produced a number of parameters (Fig. S7, S8, S9). These parameters were used to assign the molecular formula of the candidate as C28H44O3 on the basis of ESIMS analysis [*M/Z*, 427.2 (M-H)-], NMR data (Fig. 7a) and the molecular structure (Fig. 7b). These data were consistent with the structure 5α,8α-epidioxiergosta-6,22-dien-3β-ol identified as ergosterol peroxide. The purified ergosterol peroxide was 6.8 mg in mass. We dissolved the chemical in DMSO obtaining a stock solution of concentration of 2%.

Breast carcinoma cells (MT-1) were cultured in normal serum conditions and treated with ergosterol peroxide at concentrations of 0, 0.04, 0.09, 0.14, 0.18, 0.23, and 0.28 mM (or 0, 20, 40, 60, 80 100, 120 ug/ml). Our experiments indicated that ergosterol peroxide inhibited breast cancer cell growth and induced cell death starting at the concentration of 0.04 mM and induced complete cell death at the concentration of 0.28 mM (Fig. 6c, Fig. S10a). At all concentrations of ergosterol peroxide used, we detected the formation of vacuoles. Representative vacuoles formed at the concentration of 0.23 mM ergosterol peroxide are shown (Fig. 7c, lower panel).

The GFP, miR-378M, and miR-378C cells were also cultured in normal medium containing 10% FBS, and treated with ergosterol peroxide at concentrations of 0.18, 0.23, and 0.28 mM. Ergosterol peroxide induced cell death starting at the concentration of 0.18 mM, but was especially potent at the concentration of 0.28 mM (Fig. S10b). At the concentration of 0.23 mM, ergosterol peroxide induced death of miR-378C cells more than the miR-378M cells (Fig. 8a).

To corroborate these results, we analyzed a number of cell lines for miR-378 expression and found that the human hepatocellular carcinoma cell line HepG2 cells expressed high level of miR-378 (Fig. 8b). We then transfected the cells with anti-miR-378 expression construct or a control vector to down-regulate endogenous miR-378. After Ganoderma oil treatment, we found that antagonizing endogenous miR-378 decreased the sensitivity of the cells to the induced cell death (Fig. 8c). Extensive vacuole formation was detected in the HepG2 cells treated with Ganoderma oil (Fig. 8d). Statistical analysis of cell death indicated that in all concentrations tested, reducing miR-378 in tumor cells significantly decreased sensitivity to Ganoderma oil induced cell death (Fig. 8e). The cells were also treated with the purified ergosterol peroxide. Similarly, inhibition of endogenous miR-378 reduced the sensitivity of the cells to ergosterol peroxide induced cell death (Fig. 8f).

As a steroid derivative, ergosterol peroxide has been reported and isolated from a number of organisms including fungi [39]. This compound has demonstrated anti-inflammatory [39], anti-bacterial [40], anti-atherosclerosis [41], and anti-proliferative properties in human T cells [42]. Its ability to induce death in a number of cancer cell lines shed light on its potential clinical use in cancer patients. Currently, this compound can only be isolated from lower organisms and commercial products are not available. Clinical use of this compound will be contingent upon chemical synthesis and further testing.

Usually, the effects of herbal medicine rely on the synergism of multiple compounds. After a few purification steps, the effects of herbal products usually diminishes till they are hardly detectable. During the course of purifying Ganoderma oil, we found that its anti-cancer activity increased step-by-step. As such, we were able to obtain the purified compound ergosterol peroxide, which possessed potent anti-cancer properties. Since the anti-cancer activity of the biologically active components did not increase dramatically, we anticipate that there are other compounds that also possess anti-cancer activity in the oil-based substance. Further purification will be essential to obtain more compounds from *Ganoderma lucidum* with similar properties.

## Supporting Information

Figure S1 **Cancer cells transfected with** ***miR-378*** **are C2-ceramide resistant.** miR378M, miR-378C, and GFP cells were cultured in normal medium containing 30 mM C2-ceramide for different days as indicated. Cells expressing *miR-378* are resistant to C2-ceramide-induced cell death.
(PDF)

Figure S2 **Cancer cells transfected with** ***miR-378*** **are resistant to Cytarabine.** **(a)** miR378M, miR-378C, and GFP cells were cultured in normal medium containing 56 mM Cytarabine for 1 and 3 days. On day 1, the *miR-378* expressing cells exhibited lower levels of adhesion compared with GFP cells. However, on day 3, the *miR-378* expressing cells exhibited higher rates of survival than the GFP cells. (b) The cells were also treated with Cytarabine at 40 mM for 5 days. Cells expressing *miR-378* are resistant to Cytarabine-induced cell death.
(PDF)

Figure S3 **Cancer cells transfected with** ***miR-378*** **are resistant to Methotrexate.** The cells were also treated with Methotrexate at 22 mM for 6 days. Methotrexate induced cell elongation.
(PDF)

Figure S4 **Ganoderma oil exerts stronger effect on inducing death of tumor stem-like cells.** The GFP, miR-378M, and miR-378C cells were cultured in normal medium treated with different concentrations of Ganoderma oil (prepared form the standard protocol) as indicated for 24 hours. Complete cell death was marked by an “x”. The effects of Ganoderma oil on cell death were miR-378C > miR-378M > GFP.
(PDF)

Figure S5 **Ganoderma oil exerts stronger effect on inducing death of tumor stem-like cells.** The GFP, miR-378M, and miR-378C cells were cultured in normal medium treated with different concentrations of Ganoderma oil (prepared form the standard protocol) as indicated for 48 hours. Complete cell death was marked by an “x”. The effects of Ganoderma oil on cell death were miR-378C > miR-378M > GFP.
(PDF)

Figure S6 **Effects of Ganoderma oil from different sources on tumor cell death.** (a) Breast carcinoma cells (MT1) were treated with Ganoderma oil prepared from the standard protocol (WYGO-070102) and a sample of Ganoderma oil obtained from a different source (XZL). Ganoderma oil prepared from the standard protocol produced better result in inducing tumor cell death than the sample from a different source. (b) Lymphoma cells (Jurkat) were treated with WYGO-070102 and XZL. Typical cell death induced by both products are shown.
(PDF)

Figure S7 **1H-NMR analysis.** The diagram shows record at 600 MHz in CDCl3. The candidate GL421 was analyzed by 1HNMR. The hydrogen-containning functional groups were identified by chemical shift of H. The 1HNMR spectra of GL421 showed that there was one endo-double bond, one linear olefin, and one C3-OH in GL421, which are similar to sterols.
(PDF)

Figure S8 **13C-NMR analysis.** The diagram shows record at 100 MHz in CDCl3. The candidate GL421 was analyzed by 13CNMR. The C-containing functional groups were identified by chemical shift of C. The C13NMR spectra of GL421 showed that the candidate had the same side chains as sterols. Based on this information, it was deduced that there was one peroxide bridge between 79.4 (C, C-5) and 82.1(C,C-8) as they were in lower field. C-3(δ66.4) was in the higher field (δc-3 is usually 71–73 for sterol), which showed that the position of C-3 was subjected to strong γ space effect of the peroxide bridge. These data suggest that GL421 is 5,8-peroxy sterol.
(PDF)

Figure S9 **ESIMS [M/Z,427.2(M-H)-] analysis.** The candidate GL421 was assigned the molecular formula C28H44O3 based on its ESIMS (electrospray ionization mass spectrometry) and *m/z [*(mass charge ratio, *M/Z*): 427.2 (M-1)-, 409.7 (M-H20)-, 856.0 (2M-1)-].
(PDF)

Figure S10 **Effects of Ergosterol peroxide on tumor cell death.** (a) Breast carcinoma cells (MT1) were treated with ergosterol peroxide at different concentrations as indicated (mM). Induction of cancer cell death by ergosterol peroxide was concentration dependent. (b) GFP, miR-378M, and miR-378C cells were treated with ergosterol peroxide at the concentrations indicated. The sensitivities of cancer cells to ergosterol peroxide was miR-378C > miR-378M > GFP cell.
(PDF)
