# Supplementary material for: Ergosterol Peroxide Isolated from Ganoderma lucidum Abolishes MicroRNA miR-378-Mediated Tumor Cells on Chemoresistance
Source: PLoS One. 2012 Aug 30;7(8):e44579. doi: 10.1371/journal.pone.0044579 (PMC3431381; doi:10.1371/journal.pone.0044579)
Supplement: Figure S1 — Cancer cells transfected with miR-378 are C2-ceramide resistant. miR378M, miR-378C, and GFP cells were cultured in normal medium containing 30 mM C2-ceramide for different days as indicated. Cells expressing miR-378 are resistant to C2-ceramide-induced cell death. (PDF) [file pone.0044579.s001.pdf]

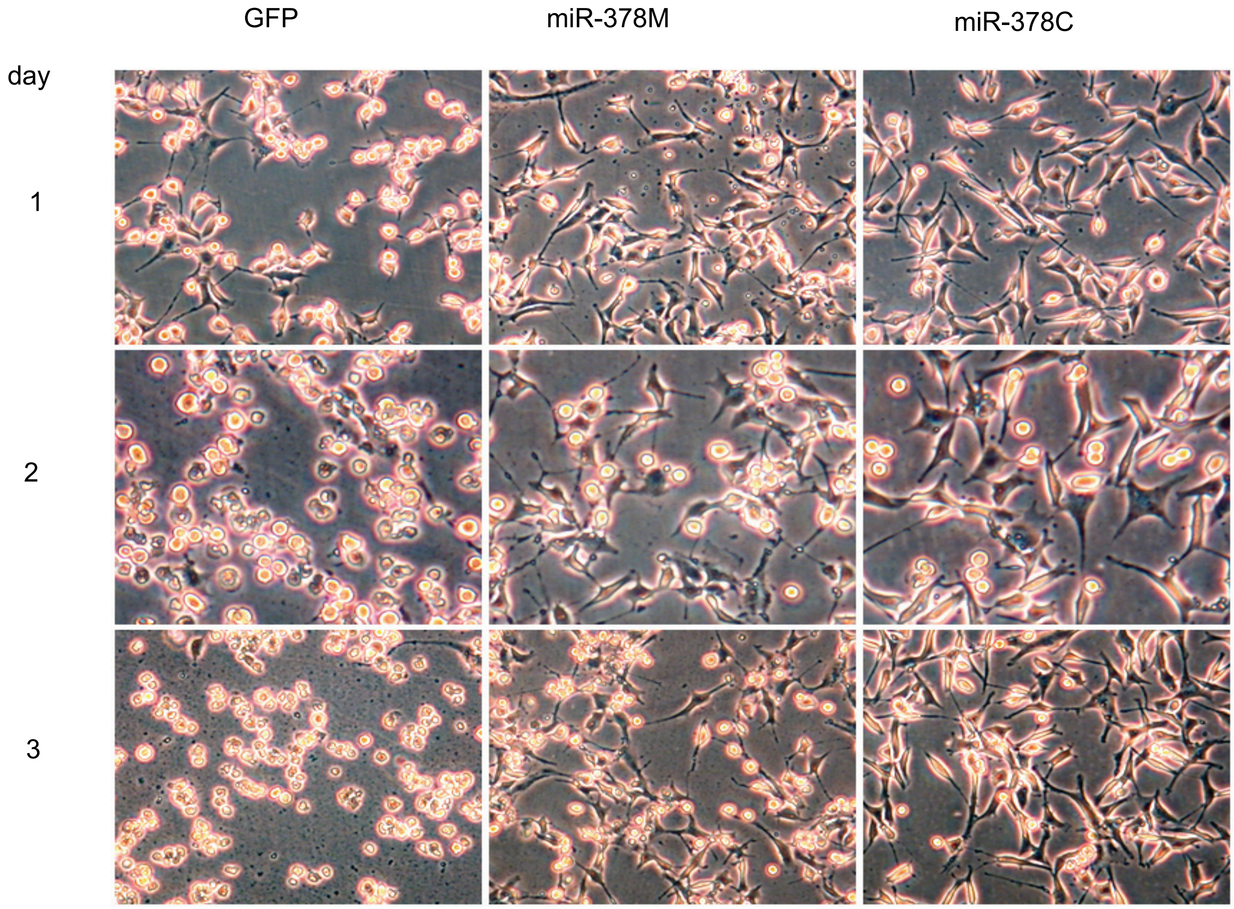

Supplementary Figure S1. Cancer cells transfected with *miR-378* are C2-ceramide resistant. miR378M, miR-378C, and GFP cells were cultured in normal medium containing 30 μM C2-ceramide for different days as indicated. Cells expressing *miR-378* are resistant to C2-ceramide-induced cell death.
