# Supplementary material for: Ergosterol Peroxide Isolated from Ganoderma lucidum Abolishes MicroRNA miR-378-Mediated Tumor Cells on Chemoresistance
Source: PLoS One. 2012 Aug 30;7(8):e44579. doi: 10.1371/journal.pone.0044579 (PMC3431381; doi:10.1371/journal.pone.0044579)
Supplement: Figure S2 — Cancer cells transfected with miR-378 are resistant to Cytarabine. (a) miR378M, miR-378C, and GFP cells were cultured in normal medium containing 56 mM Cytarabine for 1 and 3 days. On day 1, the miR-378 expressing cells exhibited lower levels of adhesion compared with GFP cells. However, on day 3, the miR-378 expressing cells exhibited higher rates of survival than the GFP cells. (b) The cells were also treated with Cytarabine at 40 mM for 5 days. Cells expressing miR-378 are resistant to Cytarabine-induced cell death. (PDF) [file pone.0044579.s002.pdf]

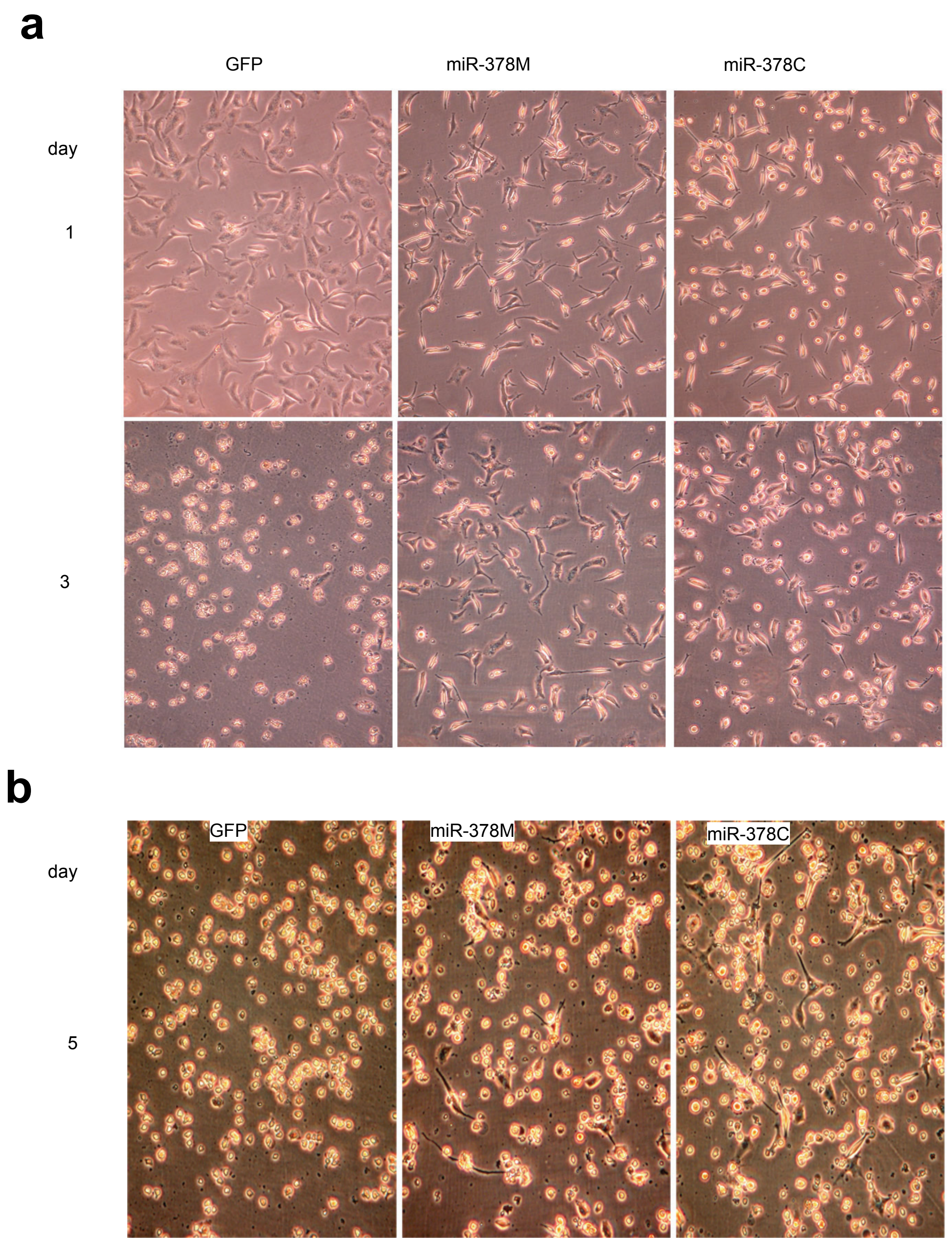

Supplementary Figure S2. Cancer cells transfected with *miR-378* are resistant to Cytarabine. (a) miR378M, miR-378C, and GFP cells were cultured in normal medium containing 56 mM Cytarabine for 1 and 3 days. On day 1, the *miR-378* expressing cells exhibited lower levels of adhesion compared with GFP cells. However, on day 3, the *miR-378* expressing cells exhibited higher rates of survival than the GFP cells. (b) The cells were also treated with Cytarabine at 40 mM for 5 days. Cells expressing *miR-378* are resistant to Cytarabine-induced cell death.
