# Supplementary material for: Ergosterol Peroxide Isolated from Ganoderma lucidum Abolishes MicroRNA miR-378-Mediated Tumor Cells on Chemoresistance
Source: PLoS One. 2012 Aug 30;7(8):e44579. doi: 10.1371/journal.pone.0044579 (PMC3431381; doi:10.1371/journal.pone.0044579)
Supplement: Figure S3 — Cancer cells transfected with miR-378 are resistant to Methotrexate. The cells were also treated with Methotrexate at 22 mM for 6 days. Methotrexate induced cell elongation. (PDF) [file pone.0044579.s003.pdf]

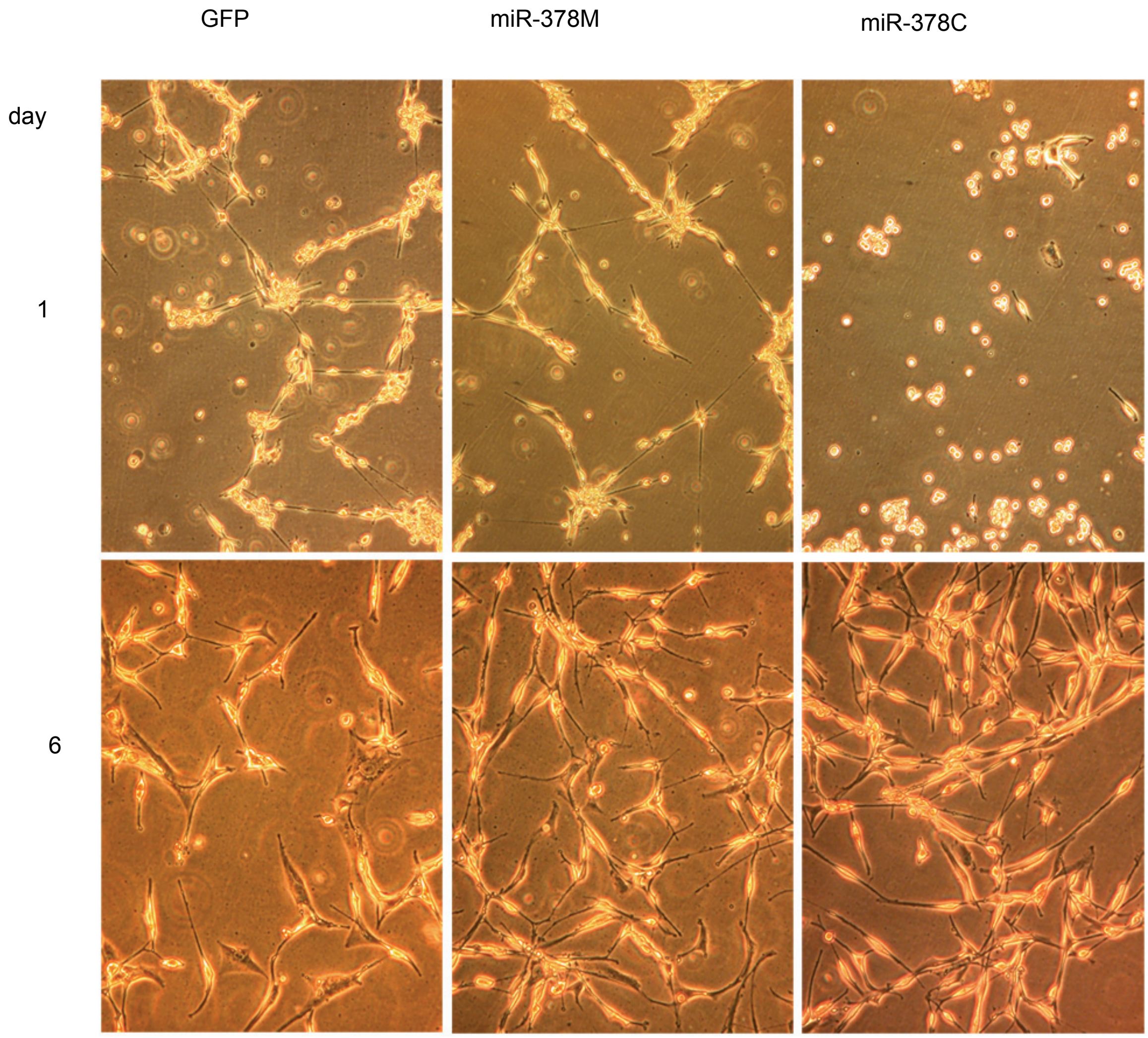

Supplementary Figure S3. Cancer cells transfected with *miR-378* are resistant to Methotrexate. The cells were also treated with Methotrexate at 22 mM for 6 days. Methotrexate induced cell elongation.
