# Supplementary material for: Ergosterol Peroxide Isolated from Ganoderma lucidum Abolishes MicroRNA miR-378-Mediated Tumor Cells on Chemoresistance
Source: PLoS One. 2012 Aug 30;7(8):e44579. doi: 10.1371/journal.pone.0044579 (PMC3431381; doi:10.1371/journal.pone.0044579)
Supplement: Figure S4 — Ganoderma oil exerts stronger effect on inducing death of tumor stem-like cells. The GFP, miR-378M, and miR-378C cells were cultured in normal medium treated with different concentrations of Ganoderma oil (prepared form the standard protocol) as indicated for 24 hours. Complete cell death was marked by an “x”. The effects of Ganoderma oil on cell death were miR-378C > miR-378M > GFP. (PDF) [file pone.0044579.s004.pdf]

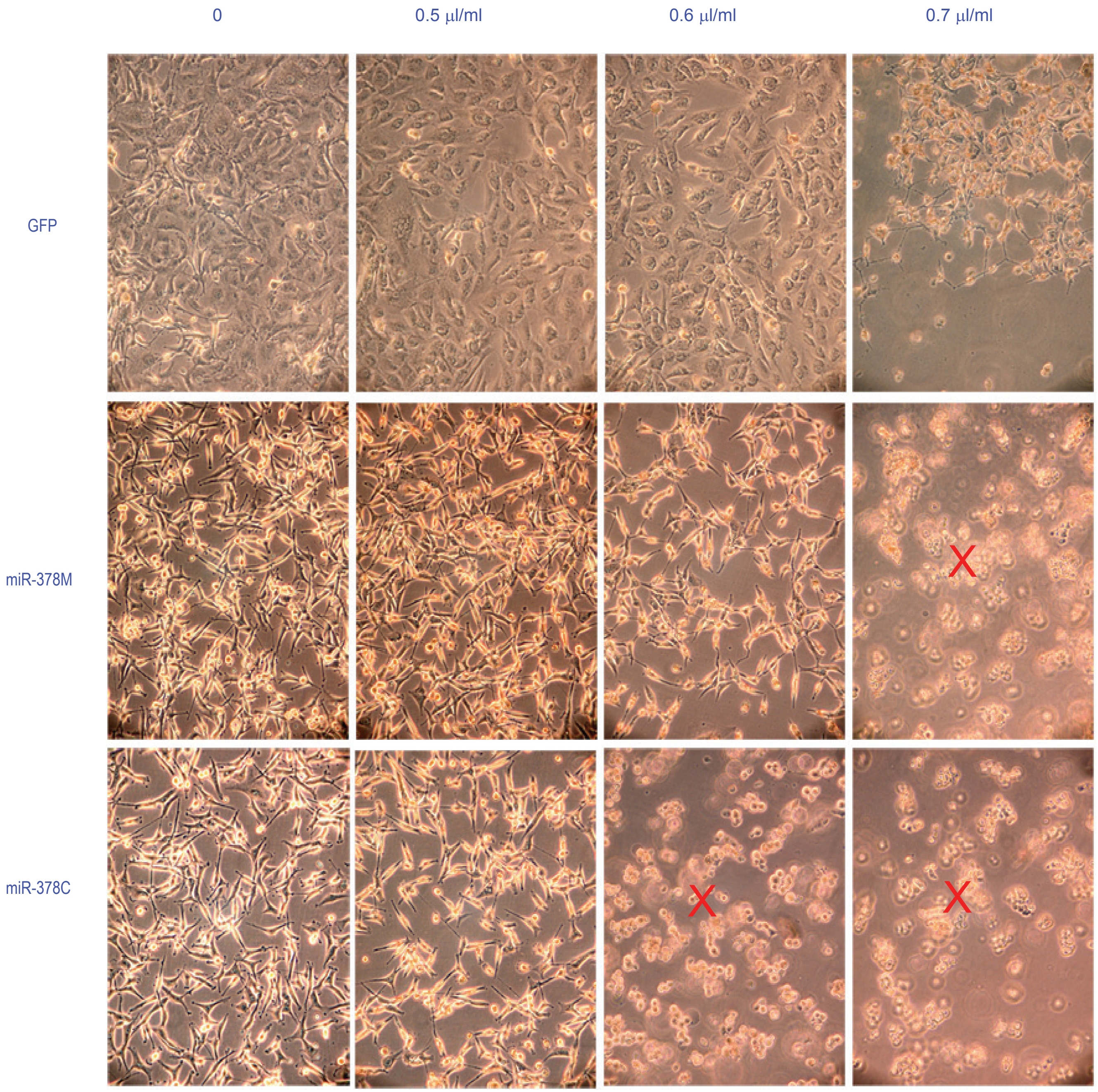

Supplementary Figure S4. Ganoderma oil exerts stronger effect on inducing death of tumor stem-like cells. The GFP, miR-378M, and miR-378C cells were cultured in normal medium treated with different concentrations of Ganoderma oil (prepared form the standard protocol) as indicated for 24 hours. Complete cell death was marked by an “x”. The effects of Ganoderma oil on cell death were miR-378C > miR-378M > GFP.
