# Supplementary material for: Ergosterol Peroxide Isolated from Ganoderma lucidum Abolishes MicroRNA miR-378-Mediated Tumor Cells on Chemoresistance
Source: PLoS One. 2012 Aug 30;7(8):e44579. doi: 10.1371/journal.pone.0044579 (PMC3431381; doi:10.1371/journal.pone.0044579)
Supplement: Figure S6 — Effects of Ganoderma oil from different sources on tumor cell death. (a) Breast carcinoma cells (MT1) were treated with Ganoderma oil prepared from the standard protocol (WYGO-070102) and a sample of Ganoderma oil obtained from a different source (XZL). Ganoderma oil prepared from the standard protocol produced better result in inducing tumor cell death than the sample from a different source. (b) Lymphoma cells (Jurkat) were treated with WYGO-070102 and XZL. Typical cell death induced by both products are shown. (PDF) [file pone.0044579.s006.pdf]

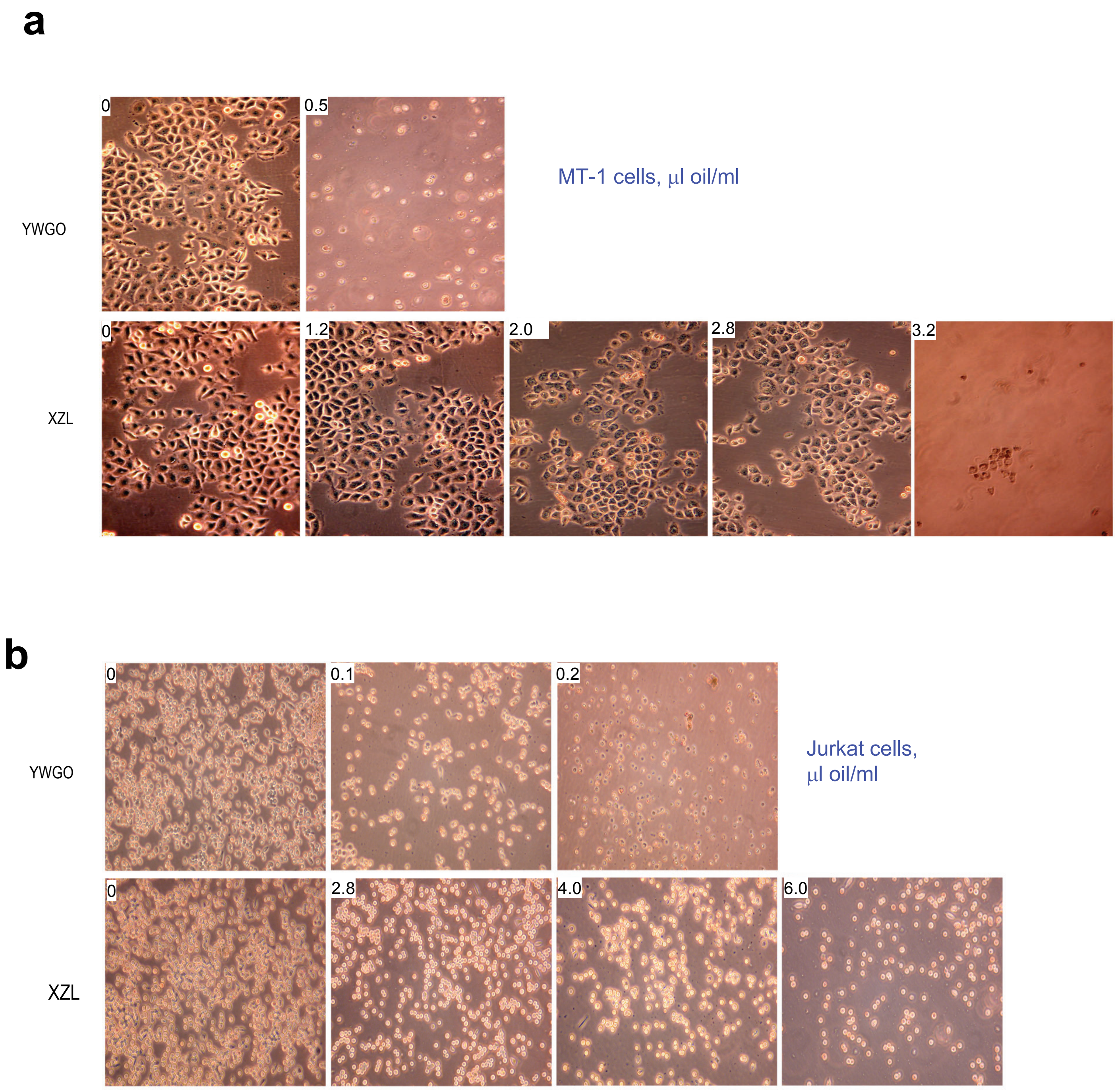

Supplementary Figure S6. Effects of Ganoderma oil from different sources on tumor cell death. (a) Breast carcinoma cells (MT1) were treated with Ganoderma oil prepared from the standard protocol (WYGO-070102) and a sample of Ganoderma oil obtained from a different source (XZL). Ganoderma oil prepared from the standard protocol produced better result in inducing tumor cell death than the sample from a different source. (b) Lymphoma cells (Jurkat) were treated with WYGO-070102 and XZL. Typical cell death induced by both products are shown.
