# Supplementary material for: Ergosterol Peroxide Isolated from Ganoderma lucidum Abolishes MicroRNA miR-378-Mediated Tumor Cells on Chemoresistance
Source: PLoS One. 2012 Aug 30;7(8):e44579. doi: 10.1371/journal.pone.0044579 (PMC3431381; doi:10.1371/journal.pone.0044579)
Supplement: Figure S7 — 1H-NMR analysis. The diagram shows record at 600 MHz in CDCl3. The candidate GL421 was analyzed by 1HNMR. The hydrogen-containning functional groups were identified by chemical shift of H. The 1HNMR spectra of GL421 showed that there was one endo-double bond, one linear olefin, and one C3-OH in GL421, which are similar to sterols. (PDF) [file pone.0044579.s007.pdf]

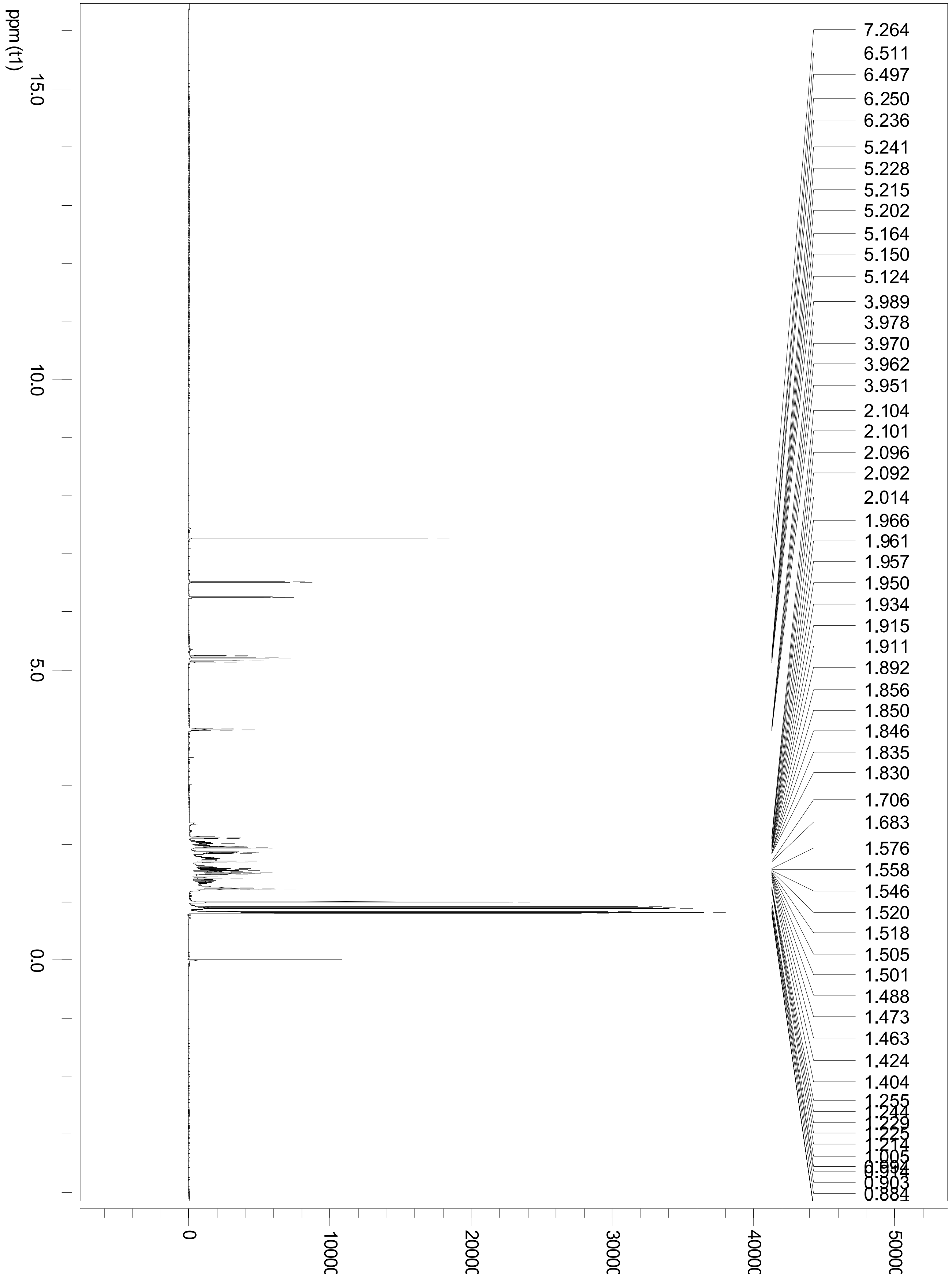

Supplementary Figure S7. 1H-NMR analysis. The diagram shows record at 600 MHz in CDCl3. The candidate GL421 was analyzed by 1HNMR. The hydrogen-containning functional groups were identified by chemical shift of H. The 1HNMR spectra of GL421 showed that there was one endo-double bond, one linear olefin, and one C3-OH in GL421, which are similar to sterols.
