# Supplementary material for: Ergosterol Peroxide Isolated from Ganoderma lucidum Abolishes MicroRNA miR-378-Mediated Tumor Cells on Chemoresistance
Source: PLoS One. 2012 Aug 30;7(8):e44579. doi: 10.1371/journal.pone.0044579 (PMC3431381; doi:10.1371/journal.pone.0044579)
Supplement: Figure S8 — 13C-NMR analysis. The diagram shows record at 100 MHz in CDCl3. The candidate GL421 was analyzed by 13CNMR. The C-containing functional groups were identified by chemical shift of C. The C13NMR spectra of GL421 showed that the candidate had the same side chains as sterols. Based on this information, it was deduced that there was one peroxide bridge between 79.4 (C, C-5) and 82.1(C,C-8) as they were in lower field. C-3(δ66.4) was in the higher field (δc-3 is usually 71–73 for sterol), which showed that the position of C-3 was subjected to strong γ space effect of the peroxide bridge. These data suggest that GL421 is 5,8-peroxy sterol. (PDF) [file pone.0044579.s008.pdf]

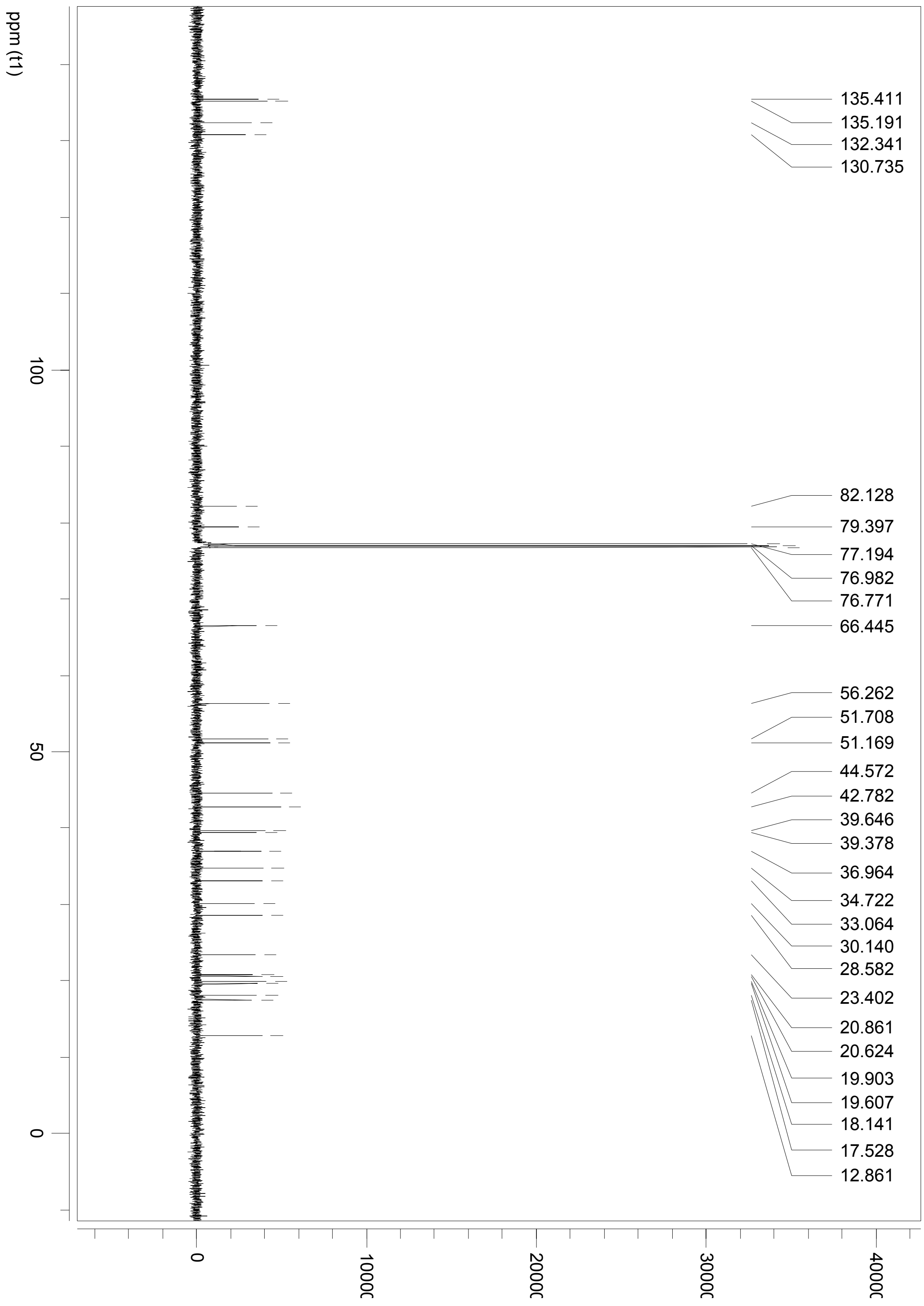

Supplementary Figure S8. 13C-NMR analysis. The diagram shows record at 100 MHz in CDCl3. The candidate GL421 was analyzed by 13CNMR. The C-containing functional groups were identified by chemical shift of C. The C13NMR spectra of GL421 showed that the candidate had the same side chains as sterols. Based on this information, it was deduced that there was one peroxide bridge between 79.4 (C, C-5) and 82.1(C,C-8) as they were in lower field. C-3(δ 66.4) was in the higher field (δ c-3 is usually 71-73 for sterol), which showed that the position of C-3 was subjected to strong γ space effect of the peroxide bridge. These data suggest that GL421 is 5,8-peroxy sterol.
