# Supplementary material for: Ergosterol Peroxide Isolated from Ganoderma lucidum Abolishes MicroRNA miR-378-Mediated Tumor Cells on Chemoresistance
Source: PLoS One. 2012 Aug 30;7(8):e44579. doi: 10.1371/journal.pone.0044579 (PMC3431381; doi:10.1371/journal.pone.0044579)
Supplement: Figure S9 — ESIMS [M/Z,427.2(M-H)-] analysis. The candidate GL421 was assigned the molecular formula C28H44O3 based on its ESIMS (electrospray ionization mass spectrometry) and m/z [(mass charge ratio, M/Z): 427.2 (M-1)-, 409.7 (M-H20)-, 856.0 (2M-1)-]. (PDF) [file pone.0044579.s009.pdf]

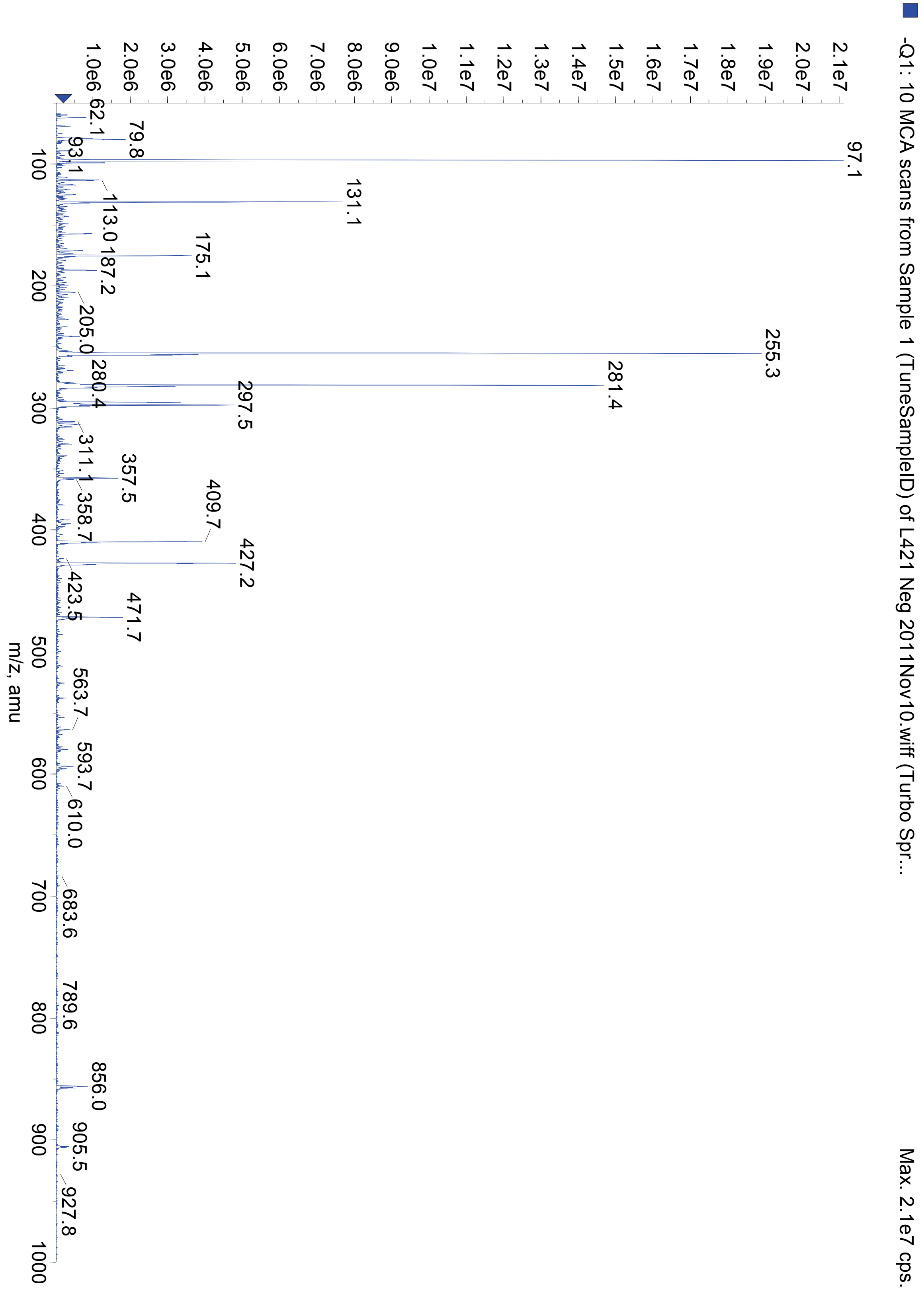

Supplementary Figure S9. ESIMS [M/Z,427.2(M-H)-] analysis. The candidate GL421 was assigned the molecular formula C28H44O3 based on its ESIMS (electrospray ionization mass spectrometry) and *m/z [*(mass charge ratio, *M/Z*): 427.2 (M-1)-, 409.7 (M-H20)-, 856.0 (2M-1)-].
