# Supplementary material for: Ergosterol Peroxide Isolated from Ganoderma lucidum Abolishes MicroRNA miR-378-Mediated Tumor Cells on Chemoresistance
Source: PLoS One. 2012 Aug 30;7(8):e44579. doi: 10.1371/journal.pone.0044579 (PMC3431381; doi:10.1371/journal.pone.0044579)
Supplement: Figure S10 — Effects of Ergosterol peroxide on tumor cell death. (a) Breast carcinoma cells (MT1) were treated with ergosterol peroxide at different concentrations as indicated (mM). Induction of cancer cell death by ergosterol peroxide was concentration dependent. (b) GFP, miR-378M, and miR-378C cells were treated with ergosterol peroxide at the concentrations indicated. The sensitivities of cancer cells to ergosterol peroxide was miR-378C > miR-378M > GFP cell. (PDF) [file pone.0044579.s010.pdf]

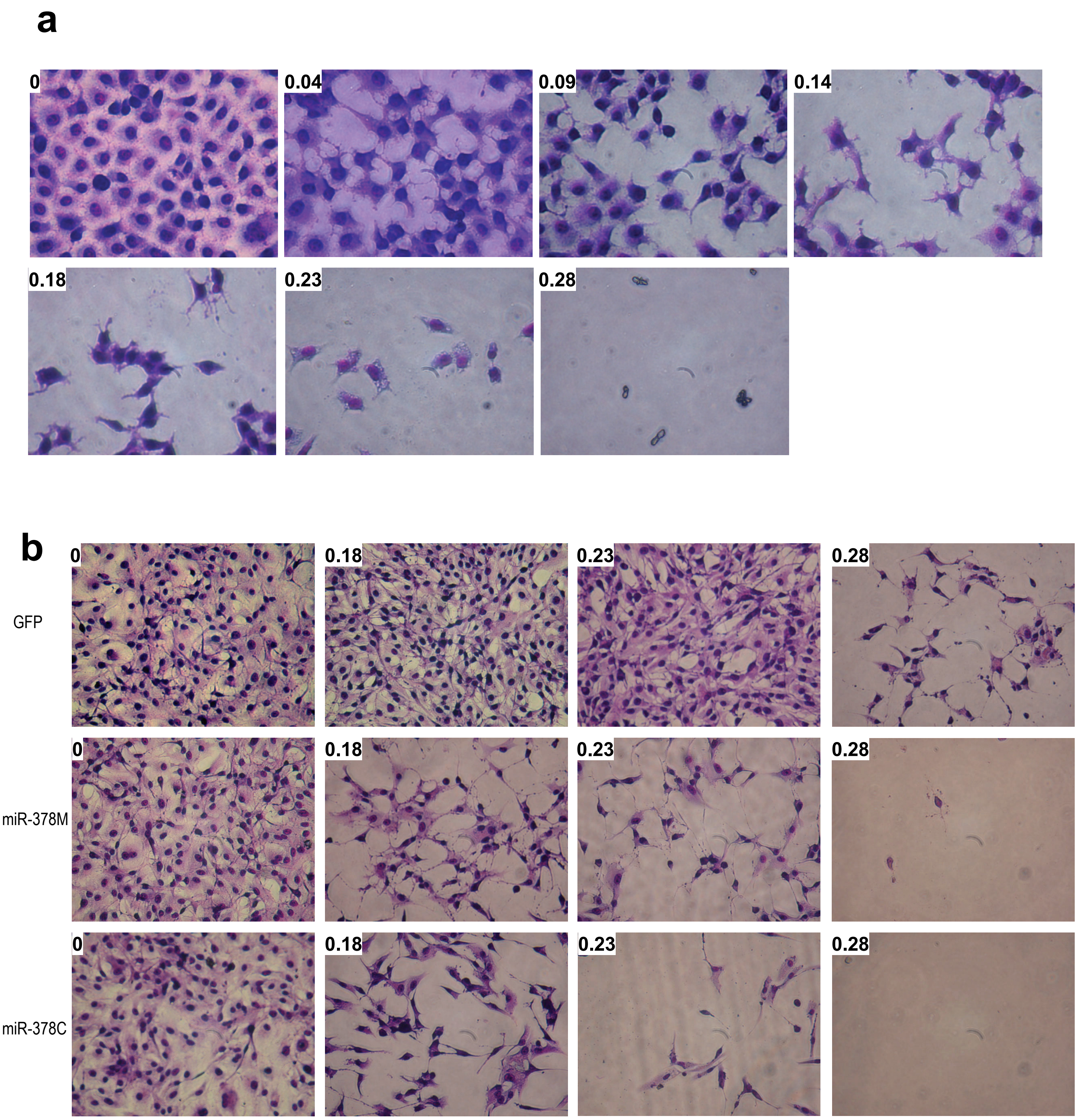

Supplementary Figure S10. Effects of Ergosterol peroxide on tumor cell death. (a) Breast carcinoma cells (MT1) were treated with ergosterol peroxide at different concentrations as indicated (mM). Induction of cancer cell death by ergosterol peroxide was concentration dependent. (b) GFP, miR-378M, and miR-378C cells were treated with ergosterol peroxide at the concentrations indicated. The sensitivities of cancer cells to ergosterol peroxide was miR-378C > miR-378M > GFP cell.
